# Mark-release-recapture studies reveal preferred spatial and temporal behaviors of *Anopheles barbirostris* in West Sulawesi, Indonesia

**DOI:** 10.1186/s13071-019-3640-3

**Published:** 2019-08-01

**Authors:** Jenna R. Davidson, Rusdiyah Sudirman, Isra Wahid, Robert N. Baskin, Hajar Hasan, Andi Muhammad Arfah, Nirwana Nur, Muhammad Yusuf Hidayat, Din Syafruddin, Neil F. Lobo

**Affiliations:** 1 Eck Institute for Global Health, University of Notre Dame, Notre Dame, IN 46556 USA; 2 Department of Parasitology, Faculty of Medicine, Universitas Hasanuddin, Makassar, 90245 Indonesia; 3 Eijkman Institute for Molecular Biology, Jakarta, Indonesia

**Keywords:** Mark-release-recapture, *Anopheles barbirostris*, Bionomics

## Abstract

**Background:**

Population density, dispersion patterns, flight distances, and survival rate of vector mosquitoes are all contributors to vectorial capacity that may be estimated in a single experimental method: mark-release-recapture (MRR). In this study, these key parameters were measured for mosquito populations in Karama, West Sulawesi, Indonesia.

**Methods:**

Two mark-release-recapture (MRR) experiments were carried out in Karama village to characterize seasonality differences, if any: wet season (December 2013, MRR1) and dry season (May 2014, MRR2). For both experiments, mosquitoes were marked according to release site/date and were released on four consecutive nights. Four sampling methodologies were utilized to enable recapture: human landing catches (HLCs), kelambu traps and barrier screens.

**Results:**

98.7% of all catches were molecularly confirmed as *Anopheles barbirostris*. During the wet season, *An. barbirostris* demonstrated no preference toward endophagy. In the dry season, *An. barbirostris* demonstrated an endophagic preference. The duration of the feeding cycle for *An. barbirostris* was determined to be 5 days during the wet season and 3.7 days during the dry season, though an anomaly likely caused the wet season feeding cycle to be overestimated. The largest percentages of recaptured mosquitoes were collected in a single site during both seasons. The only significant relationship with mosquito dispersal was site of release and recapture. Finally, dispersal rates of *An. barbirostris* frequently ranged up to 800 m (the maximum measurable distance in this study) within a single day of release.

**Conclusions:**

This study estimated key vector parameters for *An. barbirostris* an understudied species complex, in Karama, West Sulawesi, Indonesia. Despite the length of the feeding cycle, the high indoor biting rates demonstrated by *An. barbirostris* in Karama suggest that the use of IRSs and LLINs, especially during the dry season, would have a substantial impact on the panmictic *An. barbirostris* population.

## Background

The Republic of Indonesia aims to eliminate malaria by 2030 [1, 2]. Indonesia has made significant strides in recent years with over half of Indonesia’s districts declared malaria free [1]. The use of rapid diagnostic tests and treatment using effective artemisinin combination therapy in response to chloroquine resistance in Indonesia has aided the nation’s reduction in malaria [1]. Additionally, the application of interventions by the Indonesian Ministry of Health (MoH), such as distribution of insecticide-treated bed nets (ITNs), indoor-residual spraying (IRS) and larval source modification (LSM), amongst other strategies, have played large roles in the reduction of morbidity and mortality [1]. Interventions take advantage of susceptible mosquito behaviors, in this case—primarily ITNs, target mosquitos that host-seek and feed indoors. According to the World Malaria Report, 4,376,636 LLINs were distributed in 2017 to a country with a population of 264 million [3]. Furthermore, elimination efforts are complicated by complex vector and human behaviors, such as vectors’ ability to maintain disease transmission by evading interventions (behavioral resistance), and by human behaviors related to use of interventions, including exposure both outdoors in the peri-urban space and broad vocational/movement related outdoor exposure.

Difficulties also arise from the large variety of primary and secondary vector species, which includes over 20 malaria receptive anophelines in Indonesia [1, 4]. Furthermore, many of the dominant malaria vectors present in Indonesia are part of species complexes, in which cryptic morphologically indistinguishable species may have drastically different behaviors, variously effecting malaria transmission dynamics [5]. Additionally, behaviors of vectors have been shown to differ on small geographical scales, further complicating *Anopheles* control [6]. In order for Indonesia to continue advancing towards malaria elimination, knowledge about the biology and behaviors of local vectors is required to implement successful control strategies and interventions.

Population density, dispersion patterns, flight distances, and survival rates of vector mosquitoes are all contributors to vectorial capacity that may be estimated in a single experimental method: mark-release-recapture (MRR) [7–10]. In addition, MRR techniques are the most common and direct means of estimating population size [11]. There is little documentation on the feeding cycle, dispersion patterns, flight distances, and population size for *Anopheles* vectors from West Sulawesi. Feeding cycles have been reported for *Anopheles* vectors using mark-release-recapture methodologies and range from 2 to 4 days [11, 12]. A previous meta-analysis has reported *Anopheles* average maximum flight distances of 3490 m and average daily flight distance of 1040.8 m [13]. The information elucidated about these vital parameters in disease transmission dynamics makes MRR method a powerful tool.

In this study, a number of key vector parameters were measured for mosquito populations in Karama, West Sulawesi, Indonesia. The information generated from these endpoints will be used to determine potential behavioral vulnerabilities for vector control, and identify foci of vector, human or geographic risks. Our aims, for this study in Karama, were to (i) determine biting behavioral profiles for *Anopheles* mosquitoes; (ii) determine duration of *Anopheles* feeding cycles; (iii) evaluate flight dispersal of *Anopheles* mosquitoes; and (iv) estimate *Anopheles* population size.

## Methods

### Site description

**Fig. 1:**
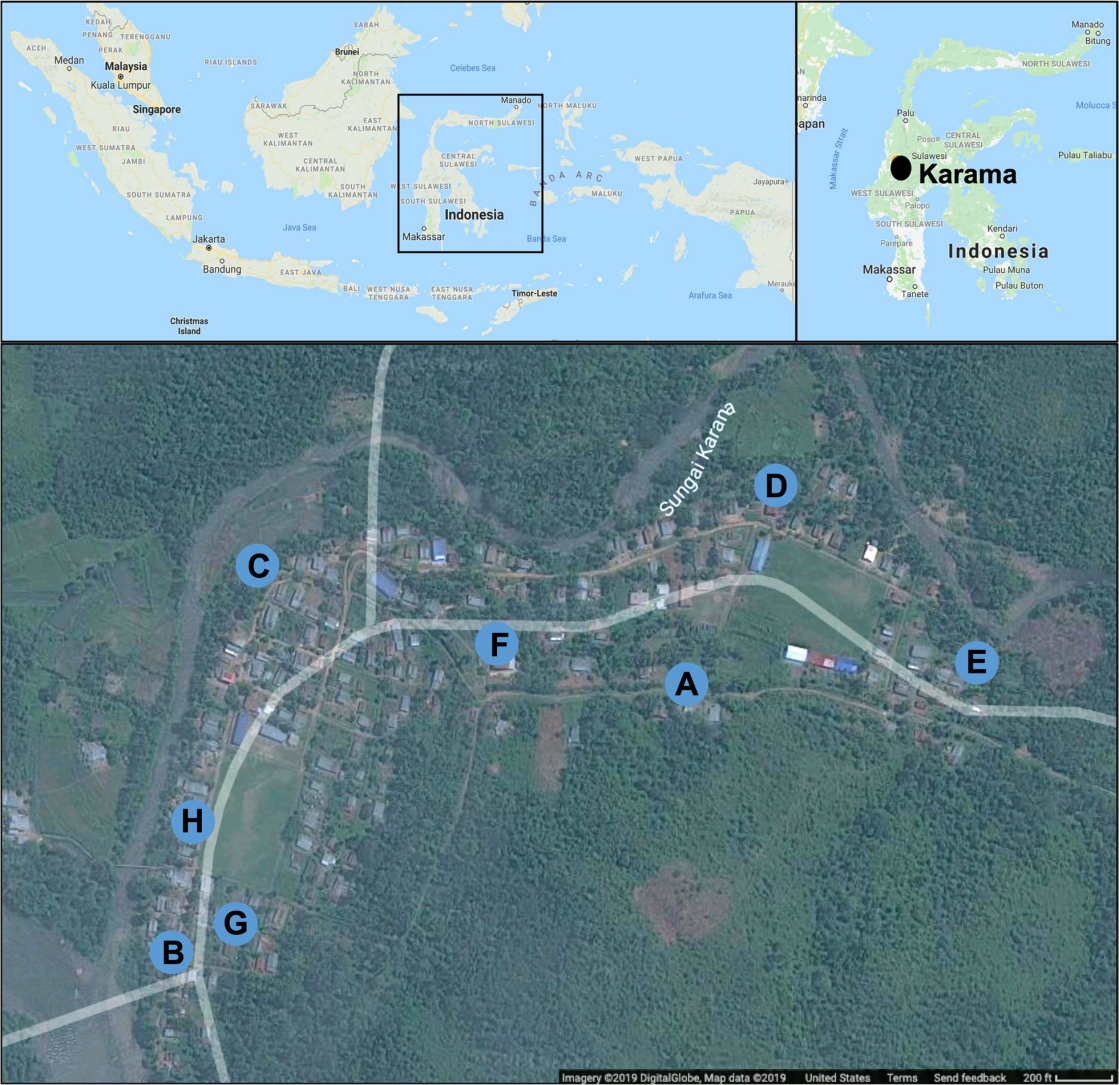
Map of Karama field collection sites. Mosquitoes were collected using kelambu traps, barrier screens, and human landing catches (inside and outside) at eight sites. Sites were located along the river Karama (Sites H, C and B), bordering the nearby forest (Sites G, E, F and A) and within the village (Sites D, G, B and F)

This study was conducted in the village of Karama, in the northwestern regency of Mamuju, West Sulawesi (Fig. 1). This isolated village, bordered by the River Karama, is partly located in the flood plain, but reaches into the foothills as houses get farther from the river. Tropical forest surrounds Karama. The main economic activity in the region is agriculture: primarily rice farming. Other popular economic activities include fishing and hunting. Houses in this area are made of wood or concrete and have thatched roofs. Low-lying houses are elevated with stilts in response to consistent flooding in the area. The open construction of these primarily wooden houses allows for free mosquito entry from all directions.

West Sulawesi has two seasons: a wet season from November to March, and a dry season from May to September. October and April are considered transition months. The mean annual rainfall in Karama is 1933 mm (maximum mean: January, 256 mm; minimum mean: August, 66 mm). The mean temperature in Karama is 27 °C (maximum mean: October, 27 °C; minimum mean: July, 26 °C) [14]. This remote area has stable, year-round malaria transmission with increased incidences during the wet season (November–March) [15].

Two mark-release-recapture (MRR) experiments were carried out in Karama village to characterize seasonality differences, if any: wet season (December 2013, MRR1) and dry season (May 2014, MRR2).

### Sampling and marking of mosquitoes

Four sampling methodologies were utilized to enable a comprehensive collection of female *Anopheles* mosquitoes: human landing catches (HLC), kelambu traps (I. Wahid, unpublished data), and barrier screens [16–18]. HLC collections were performed inside and outside of 8 houses. Collections were carried out in two-hour shifts, with a single collector indoors and a single collector outdoors in each sample house (*n* = 8). After each two-hour period, the two collectors swapped positions to reduce collector bias. The kelambu trap is an attractant-free, modified bednet trap that targets free-flying mosquitoes. The trap is separated orthogonally from each corner along the axes to give four triangular quadrants, each of which is partially open to allow for mosquito entry and the determination of mosquito flight direction. The kelambu trap is devised to make mosquito entrance to the trap easy and exit difficult. HLCs were used to target mosquitoes that feed on humans both inside and outside of houses, and net interception traps (kelambu traps and barrier screens) were utilized to capture free-flying mosquitoes that stop to rest once obstructed. The use of four sampling methodologies enabled a comprehensive, less biased, collection of mosquitoes from Karama. The same sampling method was followed for the entire experiment, pre- and post-release of mosquitoes.

Mosquito collections took place between 18.00 to 06.00 h at 8 sentinel collection sites (A-H) positioned throughout the village (Fig. 1). Sites H and B were adjacent to households as well as the river. Site G was surrounded by houses with a swamp to the south. Site C was alongside rice fields as well as houses. Sites D and F flanked houses as well as low-lying forested areas. Finally, sites E and A represented higher altitude points in the village (on hills) flanking the forest. The longest distance between two sites, D and B, was 800 m. The shortest distance between two sites, B and G, was 61 m. One sentinel house at each collection site (*n* = 8) was used for indoor and outdoor HLCs. Net traps were positioned outside, near each sentinel house. All traps were randomly rotated to multiple sites each night with only one trap per site on a given night. Additionally, traps were continually rotated past the last recorded collection day to confirm the recapture of all possible dyed mosquitoes.

Female *Anopheles* collected for were stored in cardboard cups covered with netting, each cup containing a maximum of 100 mosquitoes. Female *Anopheles* caught were blood-fed on a human volunteer who was under prophylaxis treatment (Malarone). Approximately 2.5 ml of fluorescent powder (BioQuip Products, Inc. California, USA and Glow Paint Industries, Queensland, Australia) was sifted through the netting into the cup to coat the mosquitoes. An LED UV torch (400 nm wavelength) was utilized to ensure the fluorescent powder adhered to each mosquito.

### Release of mosquitoes

For both experiments, marked mosquitoes were released on four consecutive nights between 00.00–01.00 h. Mosquitoes were marked on nights 1, 2, 3, or 4 using a different color (red, blue, yellow, and white respectively) fluorescent powder each night. Mosquitoes were released from one of four sites: site A on night 1, site B on night 2, site C on night 3, and site D on night four (Table 1). The fluorescent powder color used remained consistent by site during both studies (e.g. mosquitoes released at site A were marked with red fluorescent powder in MRR1 and MRR2) (Table 1).

**Table 1:** Mark-release-recapture release design

Night	Date released	Color marked	Site released	No. released	No. recaptured (%)
December 2013 (MRR1)					
1	November 30	Red	A	800	63 (7.9)
2	December 1	Blue	B	707	1 (0.1)
3	December 2	Yellow	C	682	157 (23.0)
4	December 3	White	D	603	13 (2.2)
May 2014 (MRR2)					
1	May 17	Red	A	225	25 (11.1)
2	May 18	Blue	B	371	3 (0.8)
3	May 19	Yellow	C	231	103 (44.6)
4	May 20	White	D	229	14 (6.1)

### Recapture of mosquitoes

Recapture of mosquitoes took place for 12 and 10 days (MRR1 and MRR2, respectively) following the release of mosquitoes. On each night following the first release, a UV light was used to identify if any captured *Anopheles* had florescent powder on them. Mosquitoes sampled from all traps were morphologically identified in the field to species [19]. Fluorescent marking color, date, time, recapture location, recapture method, and abdominal status were recorded during the sampling process.

### Human-biting profile of *Anopheles barbirostris*

The human-biting profile of *Anopheles* was described for MRR1 (wet) and MRR2 (dry) seasons using HLCs located at the eight collection sites (Fig. 1). Mean biting densities were calculated as bites/person/hour. The biting behavior of *An. barbirostris* was analyzed to estimate endophagy and nocturnal activity. Endophagy, or the preference of mosquitoes to bite indoors, was calculated as the total number of *Anopheles* collected indoors divided by the total of indoor and outdoor *Anopheles* collected [20]. The tendency for *An. barbirostris* to feed on humans during sleeping hours (21.00–05.00 h), nocturnal activity, was calculated as the total number of bites indoors plus outdoors during sleeping hours (21.00–05.00 h) divided by the total during the entire night [20]. Analysis of variance (ANOVA) was used to determine statistical preference for endophagy, nocturnal activity during sleeping hours, and the influence of collection site on HLC catches. The degrees of freedom are shown with the *F*-values associated with the factor of interest, the error, and the residual degrees of freedom of the model. Statistical analyses were carried out using GraphPad Prism 8.

### Duration of feeding cycle estimation

The length of the feeding cycle (the period between two consecutive blood meals) was estimated for *Anopheles* collected during both seasons. The mean length of the feeding cycle (U) was estimated as

$$ U = \frac{{2 \times R_{2 } + 3 \times R_{2} }}{{ R_{2} + R_{3} }} $$

where R represents the number of mosquitoes recaptured on subscript, *i* (2 and 3) days after release [20, 21].

### Net dispersal

Analysis of variance (ANOVA) was used to determine the influence of season, release site, recapture day, and abdominal status on the dispersal distance (the distance between release and recapture points in meters) by the recaptured mosquitoes. The impact of trap type was also evaluated to determine any biases in trap locations. The degrees of freedom are shown with the *F*-values associated with the factor of interest, the error, and the residual degrees of freedom of the model. Statistical analyses were performed using GraphPad Prism 8.

### Population size

Population size was estimated using the Lincoln index [22]:

$$ N = \frac{{R \sum_{d} c_{d} }}{{\sum_{d} m_{d} }} $$

where *m*_*d*_ is the number of marked mosquitoes captured on day *d* from a release of *R* marked mosquitoes on day 0, and *c*_*d*_ is the number of non-marked mosquitoes on a given day, *d*, from the population whose size is *N*. This model for estimating the mosquito population size assumes a closed population (no other villages near Karama) and no mortality (parity was not assessed). Therefore, the population size is likely overestimated.

### Molecular identification

Molecular identification was performed on approximately 15% of specimens from both MRR1 and MRR2 experiments to validate morphological identifications. Mosquitoes were individually stored in 1.5 ml microtubes over desiccant prior to molecular analysis. Molecular identification was performed using internal transcribed spacer region 2 ribosomal DNA (ITS2) [23, 24].

## Results

In Karama, Indonesia 5098 *Anopheles* mosquitoes were collected during the first mark-release-recapture (MRR1) experiment and 2879 *Anopheles* mosquitoes were collected during the second mark-release-recapture (MRR2) experiment. The majority of specimens collected during MRR1 and MRR2 were morphologically identified as *An. barbirostris* (98.7%). Of the subset of specimens selected across the longitudinal dataset, molecular identification confirmed that 98.7% were *An. barbirostris* with the remaining 1.3% consisting of *An. vagus*, *An. nigerrimus*, *An. peditaeniatus*, *An. bancroftii*, *An. kochi* and *An. sundaicus* (Table 2). Since the population was predominantly (98.7%) molecularly identified *An. barbirostris*, other species’ data were removed from all analyses other than the overall *Anopheles* population estimate in order to profile the understudied *An. barbirostris* species complex. ITS2 determined that 98.6% (*n* = 70) of the *An. barbirostris* identified were clade I, while a single mosquito was clade IV.

**Table 2:** Species identified morphologically for MRR1 and MRR2

	Kelambu trap	Barrier screen	Indoor HLC	Outdoor HLC
Wet season, MRR1				
*An. barbirostris*	38	90	59	46
*An. parangensis*	0	1	0	0
Dry season, MRR2				
*An. barbirostris*	32	27	47	35
*An. hyrcanus*	0	1	0	1
*An. nigerrimus*	1	0	0	0
*An. vagus*	0	1	0	0

### Biting profile of *Anopheles barbirostris*

During the wet season, human landing collections (HLCs) demonstrated that *An. barbirostris* had no preference toward endophagy or exophagy throughout the night (*F*_(11, 11)_ = 1.244, *P* = 0.724), and no preference in nocturnal activity during sleeping hours (21.00–05.00 h) (*F*_(3, 7)_ = 1.826, *P* = 0.460). In the dry season, *An. barbirostris* demonstrated a strong endophagic preference (*F*_(11, 11)_ = 24.28, *P* < 0.0001), but no preference in nocturnal activity during sleeping hours (21.00–05.00 h) (*F*_(3, 7)_ = 2.622, *P* = 0.265). HLC catches did not vary by collection site for both the wet and dry season (*F*_(7, 14)_ = 1.695, *P* = 0.190 and *F*_(7, 8)_ = 0.969, *P* = 0.510, respectively).

### Duration of feeding cycle for *Anopheles barbirostris*

**Fig. 2:**
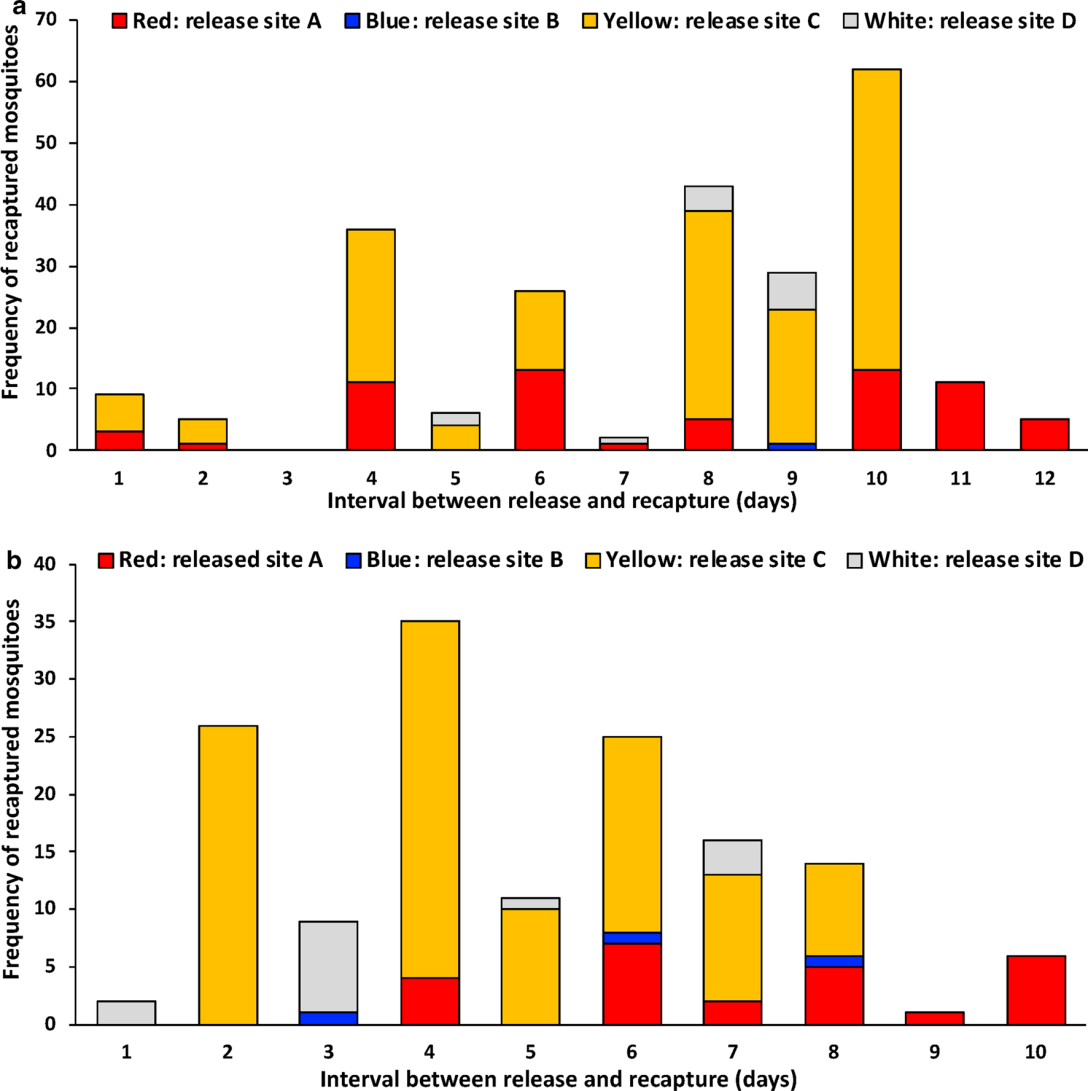
The feeding cycle length of *Anopheles* examined by a mark-release-recapture experiment, expressed as a frequency histogram of the interval of time between release and recapture for each individual mosquito for December 2013 (MRR1) (**a**) and May 2014 (MRR2) (**b**)

In December 2013 (MRR1), 2792 wild-caught female *Anopheles* of unknown chronological age were marked with fluorescent dust (a different color on each night) and released (800 on night 1; 707 on night 2; 682 on night 3; and 603 on night 4) (Table 1). Of the released mosquitoes, 234 marked female *Anopheles* were recaptured (a recapture rate of 8.4%). Of the 234 recaptured mosquitoes, 233 were *An. barbirostris* (Table 2). The highest rate of mosquito recaptured occurred on interval day 10 (26.5%) followed by days 8 and 4 (18.4% and 15.4% respectively) (Fig. 2a). The duration of the feeding cycle was determined to be 5 days for *An. barbirostris* (Fig. 2a).

In May 2014 (MRR2), 1056 female *Anopheles* of unknown chronological age were marked with fluorescent dust (a different color on each night) and released (225 on night 1; 371 on night 2; 231 on night 3; and 229 on night 4) (Table 1). Of the released mosquitoes, 145 marked females *Anopheles* were recaptured (a recapture rate of 13.7%). Of the 141 recaptured mosquitoes, 141 were *An. barbirostris* (Table 2). The highest rate of mosquito recaptured occurred on interval day 4 (24.1%) followed by days 2 and 6 (17.9% and 17.2% respectively) (Fig. 2b). The duration of the feed cycle was determined to be 3.7 days for *An. barbirostris* (Fig. 2b).

### Dispersal

**Fig. 3:**
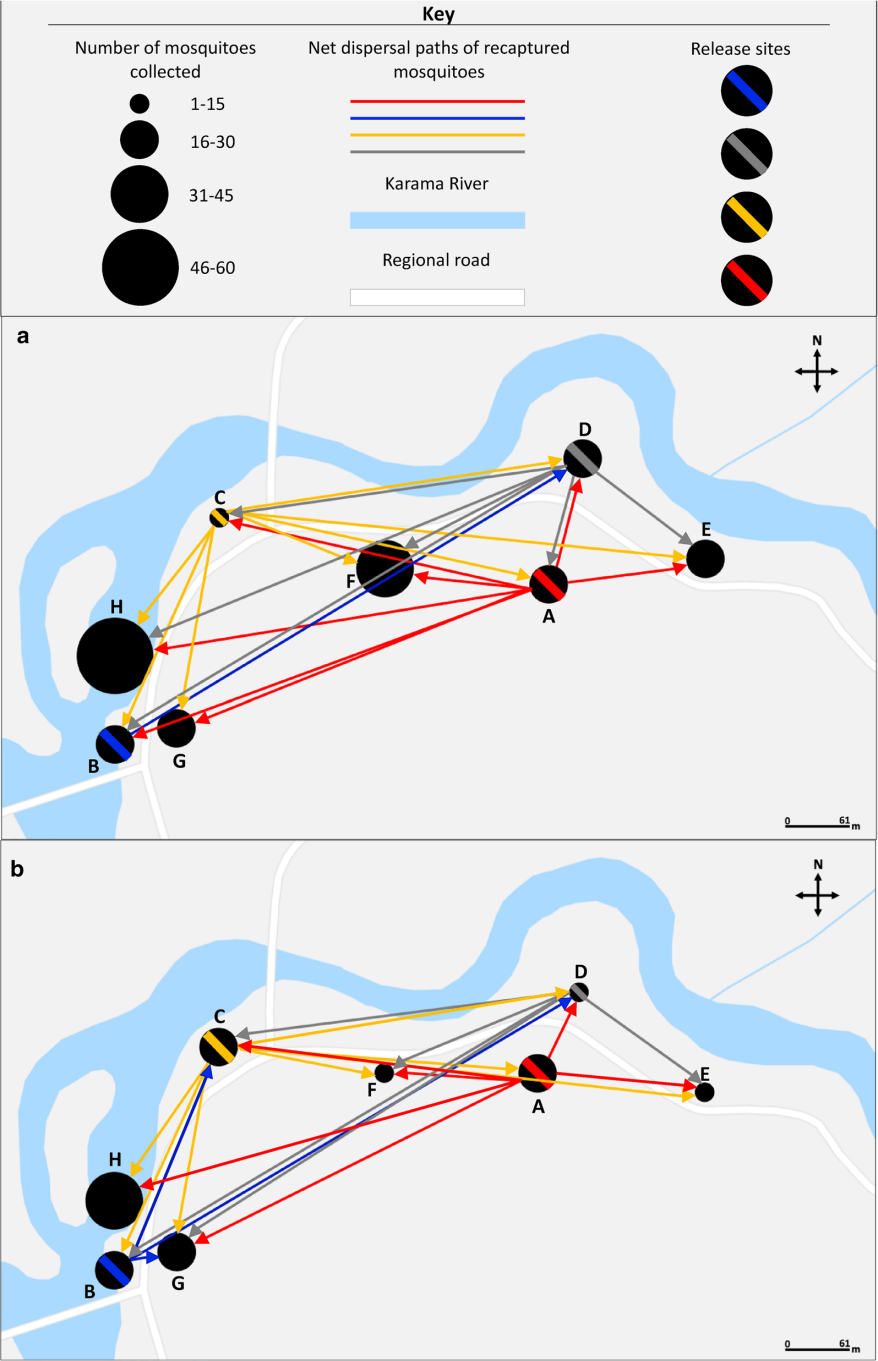
Spatial distribution of recaptured mosquitoes during the two experiments: December 2013 (**a**); May 2014 (**b**). Red, blue, yellow, and grey lines indicate the dispersal paths of mosquitoes released on day 1 (red), day 2 (blue), day 3 (yellow), and day 4 (white). Circles with red, blue, yellow, and grey diagonal lines across represent release locations

There were no statistically significant differences in mean nightly abundances between sites for both the wet season (*F*_(7, 88)_ = 0.853, *P* = 0.547) and dry season (*F*_(7, 48)_ = 0.859, *P* = 0.545) (Fig. 3). There were also no statistical differences in mean nightly abundances between trap types for both the wet season (*F*_(3, 44)_ = 0.478, *P* = 0.699) and dry season (*F*_(3, 24)_ = 0.120, *P* = 0.947).

**Fig. 4:**
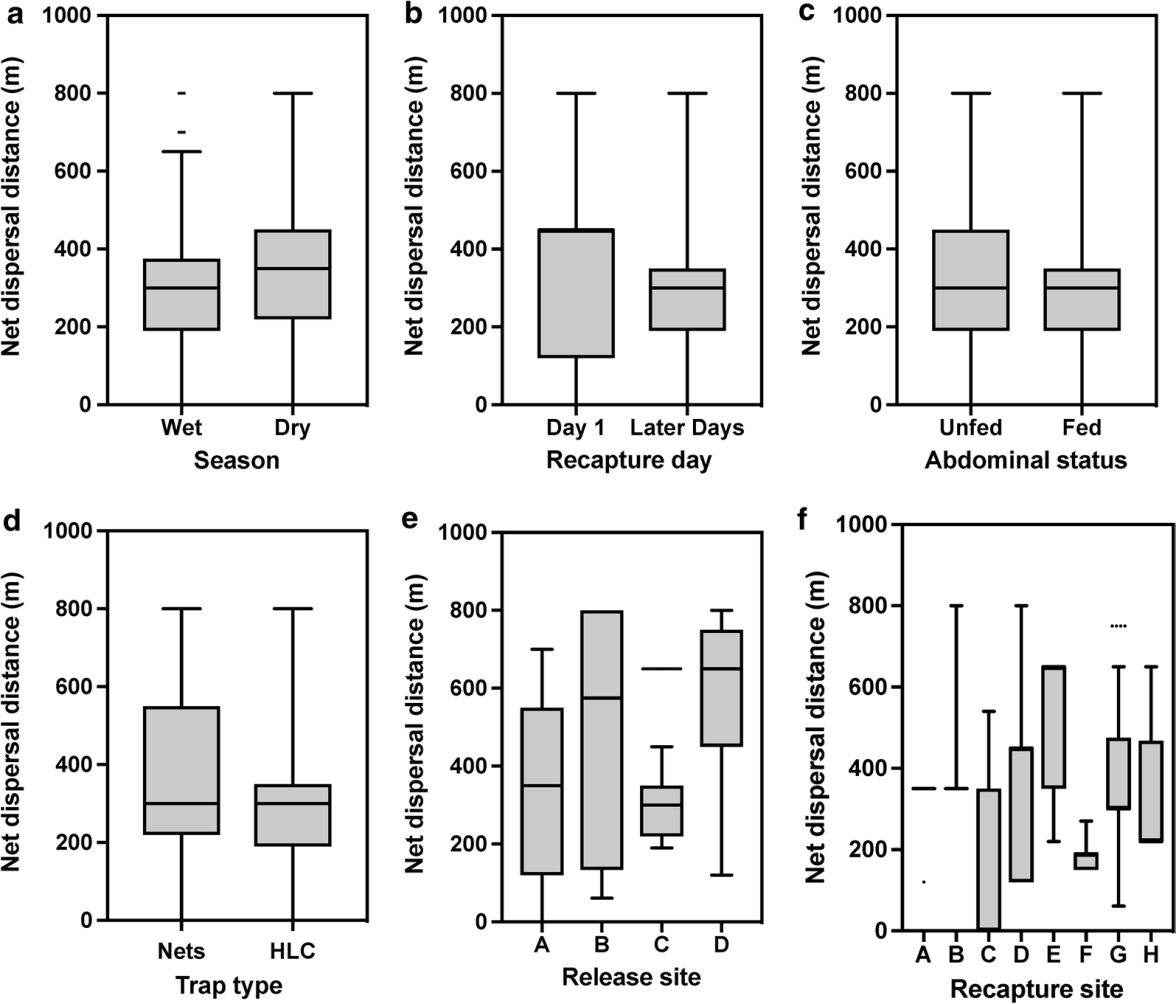
Net dispersal distances of released mosquitoes. The net dispersal distance as a function of season (**a**), time elapsed since release (**b**), abdominal status (**c**), trap type (**d**), release site (**e**) and recapture site (**f**). The bold black line is the medium distance observed. The bottom and top of the box show the first and third quartiles, while the vertical whiskers lines indicate 1.5 time the interquartile range of the data beyond which the ‘outliers’ are illustrated as individual dots

The overall distance travelled by *An. barbirostris* mosquitoes between release and recapture ranged from a minimum of 61 m to a maximum of 800 m and did not vary by season (*F*_(140, 232)_ = 1.30, *P* = 0.0785) (Fig. 4a). Additionally, overall distance travelled did not vary by recapture day (time elapsed in days between the day of release and the day of recapture) (*F*_(10, 246)_ = 1.28, *P* = 0.4891) (Fig. 4b), abdominal status (unfed *vs* fed) (*F*_(328, 47)_ = 1.26, *P* = 0.3383) (Fig. 4c) or trap type (*F*_(186, 186)_ = 1.28, *P* = 0.5614) (Fig. 4d). The strongest relationship to mosquito dispersal was site of release (*F*_(3, 370)_ = 13.49, *P* < 0.0001) (Fig. 4e) and site of recapture (*F*_(7, 307)_ = 3.94, *P* < 0.0001) (Fig. 4f). The mosquitoes released at site D had the highest mean dispersal distance, followed by the ones released at site B and then site A. Only four mosquitoes were recaptured of those released at site B.

### Population size

Population size for all *Anopheles* mosquitoes in Karama was estimated to be 58,035 during the wet season and 28,395 during the dry season.

## Discussion

This study utilized mark-release-recapture (MRR) methodology and a combination of indoor and outdoor human landing collections (HLCs) and net sampling methodologies in order to maximize trapping efficacy, collect mosquitoes associated with different behaviors, and limit biases to identify potential behavioral vulnerabilities for vector control, especially foci of vectors associated with geographical risks.

In Indonesia, biting behaviors of *Anopheles* populations vary drastically between species and islands [4]. The majority (98.7%) of molecularly identified mosquitoes at the study site were *An. barbirostris*, which is known to be a medically important vector of both malaria and filariasis in Sulawesi [25–28]. However, *An. barbirostris* is a complex of species currently known to include at least four additional species [4, 29, 30]. Previous reports have indicated strong zoophilic tendencies for this malaria vector in Indonesia [4, 31]. Furthermore, previous studies of *An. barbirostris* indicate outdoor resting and biting behaviors [32–39]. However, our study demonstrated that *An. barbirostris* mosquitoes demonstrated a strong preference for biting indoors during the dry season, and statistically similar endophagy in the wet season. This directly contradictory finding is important to understanding how biting activity and behavior can vary within a species in different locations. Notably, this discrepancy may be connected to the most abundant clade collected in this study, clade I. Molecular identification is not always performed on specimens collected and when reporting bionomic profiles of *An. barbirostris.* A previous study has reported *An. barbirostris* clades III and IV as being predominantly zoophilic [40]. Meanwhile, there is limited information on the blood-feeding preferences of clades I and II. Therefore, it is recommended that care be given when extrapolating a species’ bionomic information across all members of the complex, though future research would have to confirm whether the findings of this study are replicable across similar locations. This is a promising find, as distributions of LLINs for use, especially in the dry season, may be an appropriate intervention for the panmictic *An. barbirostris* population in locations similar to Karama.

The feeding cycle is defined as the period between blood-feeding events. In this study, the feeding cycle length of *An. barbirostris* was estimated to be 5 days during the wet season, MRR1 and 3.7 days during the dry season, MRR2. The feeding cycle length during the dry season is similar with previous estimated reports of *An. barbirostris* from Cambodia, in which feeding cycle lengths from four villages were reported to be between 3.71–4.0 days [41]. The longer than expected feeding cycle estimate during the wet season (5 days) is influenced by mosquitoes not being collected on day 3 (Fig. 2a) and this anomaly likely resulted in an overestimation of feeding cycle length during the wet season. A long feeding cycle, like that of *An. barbirostris* in this study, may reduce this species complex’s likelihood to encounter interventions such as IRSs and LLINs. Despite the longer than expected feeding cycle, the high indoor biting rates demonstrated by *An. barbirostris* suggest that the use of IRSs and LLINs, especially during the dry season, would have a substantial impact on the panmictic *An. barbirostris* population.

Only four mosquitoes were recaptured of those released at site B. Lower survival rates may have been due to the effects of dye color [42]. However, it is more likely that mosquitoes released at site B may have been more likely to emigrate south into the adjacent forest to exploit preferred hosts (macaques and birds) [43–45] and diverse ecological niches (ground pools and tree holes) [46, 47]. Future studies of *An. barbirostris* spatial dynamics will be necessary to discern the factors influencing mosquito dispersal in locations similar to Karama. Identifying preferred habitats for *An. barbirostris* that can be generalized to similar locations has direct implications for vector control and interventions.

Measurements of overall dispersal distance for *An. barbirostris* allowed for the assessment of factors that may be important to mobility. In this study, many *An. barbirostris* mosquitoes were found to have dispersed 800 m in overall distance from their release location. However, overall dispersion measures the distance, in a direct line, from release site to recapture site, and therefore almost certainly underestimates actual flight distance for mosquitoes. Dispersal measures were not influenced by season (Fig. 4a), recapture day (mosquito dispersal distance varied widely even within a single recapture day), trap type, or abdominal status. In this study, relationship for dispersal was strongly related to release and recapture location. In other words, site D had the highest dispersal rate. However, this observation is most directly due to location of site D, which is located on the northeast site of the village, with the farthest average distance from other recapture locations. Ultimately, the fact that the only established relationship to dispersal distance was site locations most likely means that *An. barbirostris* mosquitoes were dispersing at a distance greater than could be evaluated by the proximity of the traps in this study, especially considering many recaptured *An. barbirostris* were caught at the maximum measurable distance (800 m) within a single day of release. To overcome this limitation of this research, future studies that aim to determine the dispersal distance of *An. barbirostris* should include distances greater than 800 m to more accurately evaluate the dispersal rate of *An. barbirostris* and associated behaviors to dispersal rates.

## Conclusions

This study estimated key vector parameters for *An. barbirostris* an understudied species complex, in Karama, West Sulawesi, Indonesia. *An. barbirostris* demonstrated high levels of indoor biting activity: equally preferring indoor biting in the wet season and strongly preferring indoor biting in the dry season. Previous description of *An. barbirostris* documents the complex to be primarily exophagic, so this directly contradictory finding is important to understanding the extent to which biting activity and behavior can vary within a species complex in different locations. *Anopheles barbirostris* demonstrated a longer-than-expected feeding cycle for an *Anopheles* mosquito, which may reduce this species complex’s likelihood to encounter interventions such as IRSs and LLINs. However, the high indoor biting rate demonstrated by *An. barbirostris* in Karama suggests that the use of IRSs and LLINs, especially during the dry season, would have a substantial impact on the panmictic *An. barbirostris* population. Finally, dispersal rates of *An. barbirostris* frequently ranged up to 800 m (the maximum measurable distance in this study) within a single day of release, meaning future studies hoping to establish dispersal distance to behavioral objectives for the species complex should consider including capture locations of greater distance. The information generated from these endpoints will be used to determine potential behavioral vulnerabilities for *An. barbirostris* control.

## Data Availability

Data supporting the conclusions of this article are included within the article. The datasets used and/or analyzed during the present study are available from the corresponding author upon reasonable request.

## Abbreviations

ITN: insecticide-treated net
LLIN: long-lasting insecticidal net
IRS: indoor residual spraying
HLC: human landing collection
MRR: mark-release-recapture
ANOVA: analysis of variance
